# The positive impact of silicon on the yield and quality of tobacco

**DOI:** 10.3389/fpls.2025.1641798

**Published:** 2025-08-04

**Authors:** Mengjie He, Qiang Li, Ya Ma, Pengtao Zhou, Kang Kang, Boran Wu

**Affiliations:** ^1^ College of Resources, Hunan Agricultural University, Changsha, Hunan, China; ^2^ College of Agriculture, Hunan Agricultural University, Changsha, Hunan, China

**Keywords:** silicon, tobacco, growth, nutrient use efficiency, economic benefits

## Abstract

Silicon (Si) is widely used in agricultural crop practices. However, the effects of varying Si application rates on tobacco growth and quality remain unclear. Therefore, this study applied four different Si concentrations, i.e., 0, 750, 1500 and 3000 kg/ha of Si (S0, S50, S100 and S200), examined the impact of different Si concentrations on tobacco (*Nicotiana tabacum* L. ‘Yunyan 87’) growth, nutrient utilization, and economic quality under field conditions. The results demonstrated that Si application significantly improved tobacco growth, the biomass significantly increased by 19.5%-26.53%; during button stage, the plant height significantly increased by 15.38%-19%. Si also enhanced nutrient use efficiency, particularly for nitrogen and potassium. The utilization efficiency of N and K fertilizer were significantly increased by 27.42%-43.71% and 40.25% - 44.63%, respectively. Furthermore, Si improved leaf physical properties, enhancing single-leaf weight and leaf area, while reducing leaf density and midrib ratio, optimizing leaf quality by improving the sugar-alkali ratio and potassium-chloride balance. Notably, the reducing sugar content in upper leaves increased by 15.21% with S50 treatment, while the chlorine content in middle leaves was decreased by 11.11% with S100. Additionally, among all treatments, S50 achieved the highest proportion (94.75%) of medium and high-quality tobacco leaves, along with a 15.70% increase in yield and a 30.76% boost in output value compared to S0. However, excessive Si application (3000 kg/ha) negatively affected quality, increasing nicotine levels and disrupting the sugar-alkali ratio, which elevate leaf irritancy. In conclusion, moderate Si application (750–1500 kg/ha) is an effective strategy for enhancing tobacco yield and quality, offering a sustainable approach to optimize cultivation practices.

## 1 Introduction

Tobacco (*Nicotiana tabacum* L.) as a widely cultivated economic crop, is extensively grown in Brazil, China, the United States, and India (Lisuma et al., 2023). As a globally traded crop, tobacco yields and quality is an important indicator of tobacco plant that determines farmers’ income and tobacco market prices (Yan et al., 2025). Therefore, the yield and quality of tobacco leaves deserve special attention.

Silicon (Si), as a beneficial element for plants, is widely used in rice, tomatoes, sugarcane and other high Si demanding crops (Yan et al., 2023; Fan et al., 2018; Verma et al., 2020a). Researchers found that Si promoted the root development of plants, increased photosynthetic efficiency and the dry matter mass, thereby increased yields (Ji et al., 2017). Besides, Si improved the stress resistance of plants, such as salt stress, heavy metal toxicity, drought, high temperature, and ultraviolet radiation (Verma et al., 2023; Verma et al., 2020a; Ghosh et al., 2022; El-Saadony et al., 2022). And Si effectively reduced crop lodging and improved insect resistance by enhancing the mechanical strength such as stems and cell walls, especially under adverse weather conditions (Kumari et al., 2022; Khan et al., 2021; Pavlovic et al., 2021). In addition, the application of Si not only impacted plants, but also had a significant impact on soil physicochemical properties and microbial community structure (Artyszak, 2018). Si application altered the chemical composition of soil minerals by promoting the formation of new clay minerals (Thakral et al., 2024). These clay minerals exhibited a high specific surface area, enabling the adsorption of water, nutrients nitrogen (N), phosphate (P), potassium (K), aluminum (Al), and heavy metals through both physical and chemical mechanisms, which increased soil available Si content, reduced soil heavy metal toxicity, regulated soil pH value, enhanced soil water retention and permeability, thereby improved soil quality, and promoted nutrient use efficiency (Souri et al., 2021; Song et al., 2022; Schaller et al., 2021).

However, it was reported that excessive application of Si may have negative effects, which lower soil pH, leading to soil acidification, and affected soil microbial activity (Schaller et al., 2021). Besides, Si competes with other nutrients such as N, P and K for absorption, and excessive application may lead to nutrient imbalance, affecting normal plant growth (Coskun et al., 2019). Researchers have found that, the demand for Si varies among different plants. For example, rice is a high Si demanding crop with a critical concentration of 30-50g/kg, while the Si critical concentration of tomatoes is 5-8g/kg (Thakral et al., 2024).

In addition, current researches on Si fertilizer predominantly focuses on field crops harvested for seeds and fruits, as well as vegetables, whereas tobacco cultivation primarily targets leaf production (Thakral et al., 2024). However, there is limited research on the effects of Si fertilizer on tobacco. The appropriate application amount of Si fertilizer on tobacco is not clear, and more research is needed to explore the appropriate application amount of Si fertilizer in tobacco agricultural production. Based on this, we conducted field experiments on *Nicotiana tabacum* L. ‘Yunyan 87’, to investigate the influence of different rate of Si fertilizer on tobacco growth, yields and quality, five experimental treatments were established in this study: CK (without fertilization), S0 (control; conventional fertilization), S50 (application of Si fertilizer at 750 kg/ha), S100 (application of Si fertilizer at 1500 kg/ha), and S200 (application of Si fertilizer at 3000 kg/ha).The agronomic traits, physical characteristics, quality characteristics, yield, output value, average price, nutrient accumulation and fertilizer utilization rate of N, P, K, were assayed in this study. This study to provide a scientific basis for rational Si fertilizer utilization in tobacco cultivation, and practical fertilizer usage guidelines for tobacco farmers to improve tobacco quality and overall market value.

## 2 Materials and methods

### 2.1 Experiment design and field location

**Figure 1:**
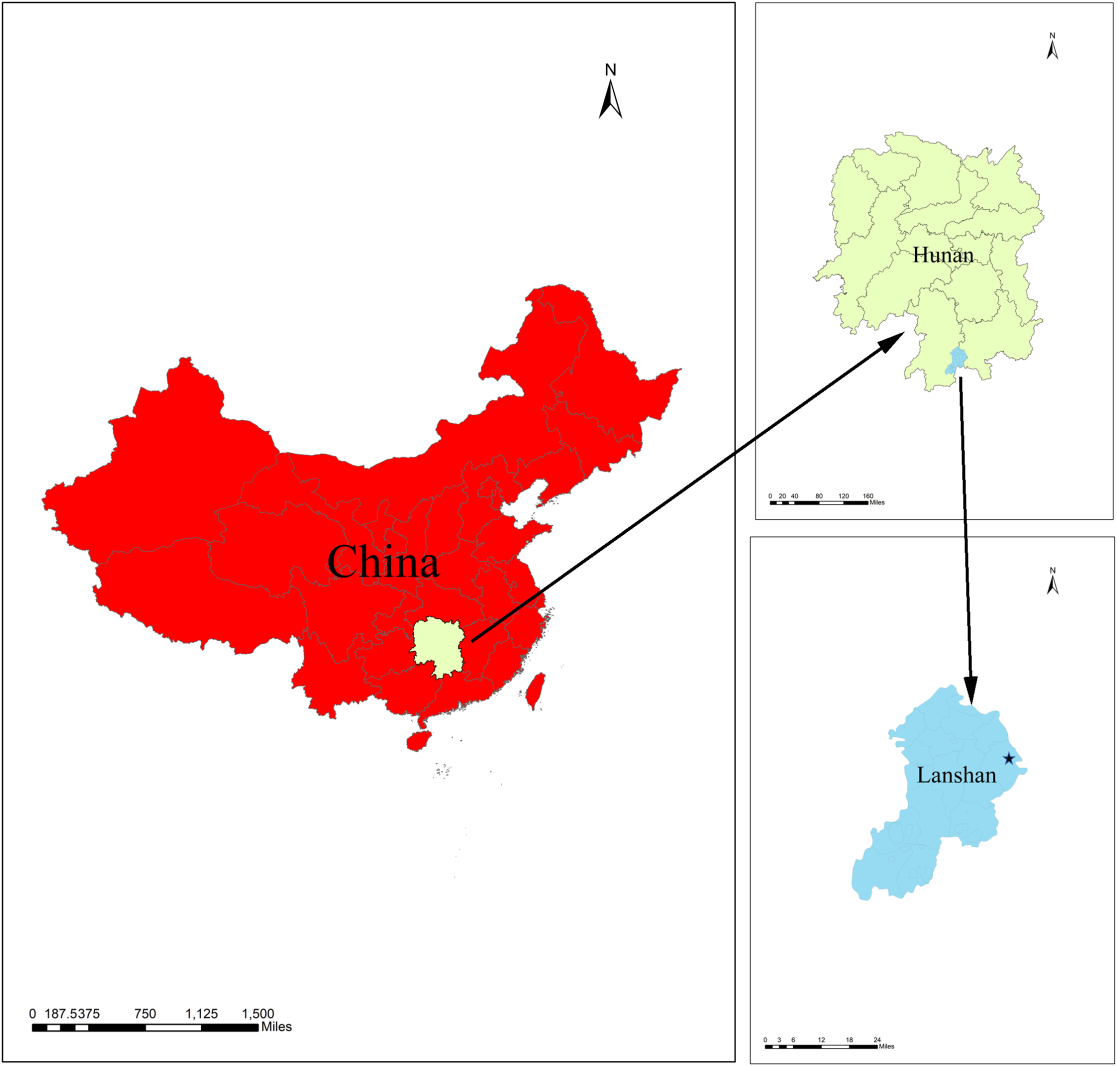
The experimental site location. The pentagram mark in the picture indicates the location of the experimental field.

**Figure 2:**
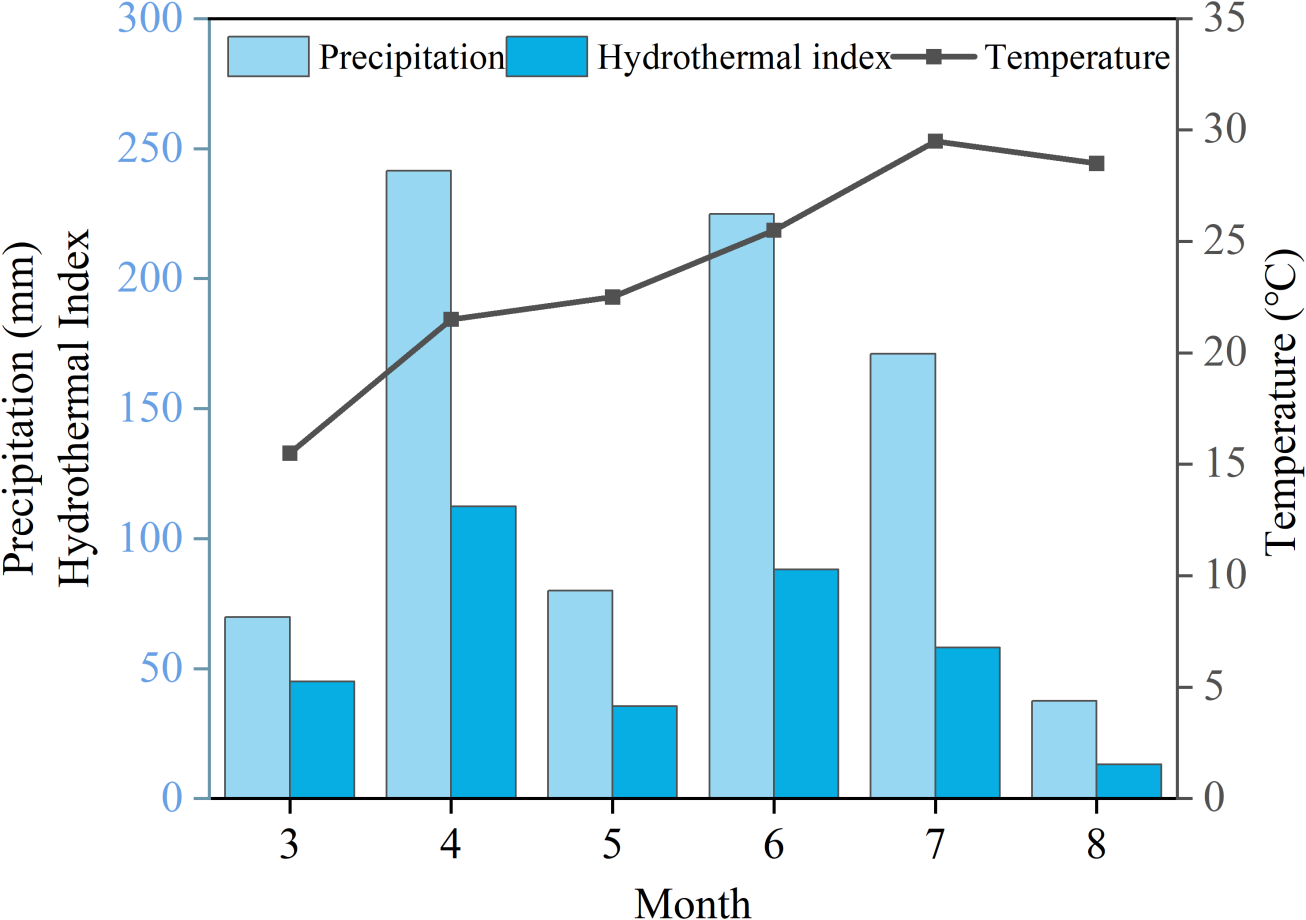
Climate data from March to August. On the left are precipitation and hydrothermal index, and on the right are temperature.

The tobacco variety (*Nicotiana tabacum* L. ‘Yunyan 87’) was used in this study, which has good disease resistance, coordinated dehydration and drying, and is easy to bake. The field experiment was conducted in Lanshan County, Yongzhou City, Hunan Province, China (25.18°N, 112.18°E) from March to August 2024. The specific location is shown in **Figure 1**. The annual average temperature in this area is 18°C to 19 °C, the annual precipitation is 1400 to 1600 millimeters, and the annual sunshine hours are 1400 to 1600 hours. The climate data from March to August, including temperature, precipitation and hydrothermal index are shown in **Figure 2**. The basic physicochemical properties and the microelement content of the soil (0–20 cm) were as follows: pH, 6.73; organic matter (OM), 65.97 mg/kg; alkali-hydrolysable nitrogen (AN), 229.10 mg/kg; available phosphorus (AP), 102.81 mg/kg; available potassium (AK), 355.85 mg/kg; total nitrogen (TN), 3.64 g/kg; total phosphorus (TP), 2.42 g/kg; total potassium (TK), 6.63 g/kg; chloride ion (Cl^-^), 5.74 mg/kg; boron (B), 3.69 (mg/kg); available SiO_2_, 6.98 mg/kg; manganese (Mn), 38.92 mg/kg; iron (Fe), 357.89 mg/kg; nickel (Ni), 3.21mg/kg; copper (Cu), 2.08 mg/kg; zinc (Zn), 9.14 mg/kg.

Tobacco was transplanted on March 27, 2024, the lower leaves were harvested on June 26, 2024, the middle leaves were harvested on July 15, 2024, and the upper leaves were harvested on August 1, 2024. The apical part was excised 2–3 weeks prior to floral anthesis, and basal leaf removal was implemented when 30–50% of the central inflorescence had reached bloom stage, ultimately retaining 18 functional leaves per plant through systematic defoliation. In order to ensure the growth and development of plants, high ridge cultivation was adopted during transplantation, covered with plastic film. During the period of plant growth, leaf diseases and leaf eating pests were controlled, and solar insecticidal lamps were installed in the field.

Five experimental treatments were established, CK is no fertilization, S0 is normal fertilization, S50 is normal fertilization+750 kg/ha Si fertilizer, S100 is normal fertilization+1500 kg/ha Si fertilizer and S200 is normal fertilization+3000 kg/ha Si fertilizer. The plant spacing and row spacing are 50 × 120 cm. Three replicates were set up for each treatment, with each replicate area of 4 m × 8.4 m, and each treatment was randomly distributed.

The Si fertilizer used contains SiO_2_ 50%, CaO 12%, MgO 0.7%, Fe_2_O_3_ 2.3%, organic matter 10%, humic acid 6% and other trace elements 3%. Fertilizer application refers to **Table 1**. The nutrient input of each treatment was 185.4 kg/ha of N, 127.5 kg/ha of P_2_O_5_, and 430.5 kg/ha of K_2_O.

**Table 1:** Fertilizer application.

Time	Fertilization type	Application ratio
5 days before transplanting	Base Fertilizer (1st)	70%
3 days after transplanting	Base Fertilizer (2nd)	30%
25 days after transplanting	Topdressing	Conventional fertilization

Fertilizer ingredients: tobacco specific base fertilizer (8-10-11); seedling fertilizer (20-9-0); tobacco specific topdressing (10-0-32); potassium sulfate (K_2_O=52.0%, Cl=1.5%, S=17.0%); silicon fertilizer (SiO_2_ = 50%).

### 2.2 Collection of tobacco and soil samples

Soil samples ranging from 0–20 cm were gathered from 10 random sites within each plot on March 15, 2024 and after visible plant residues and stones were removed. The soil samples were air-dried, pulverized, and sieved through 2 mm and 0.0149 mm screens for subsequent analysis, including pH, OM, HN, AP, AK, TN, TP, TK, Cl^-^, B, available SiO_2_, Mn, Fe, Ni, Cu, Zn.

At the flower budding stage and button stage of tobacco, 10 plants with consistent growth and no pests or diseases were randomly selected from each plot. The 3rd, 6th, and 9th leaves were selected from top to bottom for SPAD value measurement. Agronomic traits such as plant height (cm), stem circumference (cm), effective leaves (leaves/plant), maximum leaf length (cm), maximum leaf width (cm), and maximum leaf area (cm²) were recorded for each treatment. At the maturating stage of tobacco, the harvested mature tobacco leaves were baked and divided into three parts, including upper leaves (the 1st to 6th leaves), middle leaves (the 7th to 12th leaves), and lower leaves (the 13th to 18th leaves). Physical properties including leaf length (cm), leaf width (cm), leaf area (cm²), single leaf weight (g), density of leaf(g/m^2^), leaf moisture content (%), leaf midrib proportion (%), thickness (mm) and opening degree (%) were measured. After physical property assessment, economic traits such as yields, output value, and average price, proportion of high-quality tobacco leaves and proportion of medium to high-quality tobacco leaves were evaluated. Then, the tobacco samples were ground into fine powders for chemical composition analysis, including total sugar, reducing sugar, total N, nicotine, K, chlorine, starch, and protein. At the same time, 10 plants with consistent growth and no pests or diseases were randomly selected from each plot. The plants were dug out, cleaned, and divided into five parts: roots, stems, upper leaves, middle leaves, and lower leaves. Biomass and nutrient content for each part were subsequently measured.

### 2.3 Measurement methods

#### 2.3.1 Soil

The soil samples were measured using the method as described by Schaller et al. (2021) and Islam et al. (2023). In brief, the soil pH was determined using a pH meter (ORION3STAR, Thermo Fisher Scientific, USA) in a 1:25 (w/v) paste with deionized water. OM and AN were quantified using high-temperature potassium dichromate oxidation and the alkaline hydrolysis diffusion method, respectively. AP was extracted using the Olsen method, and readily AK was extracted using the ammonium acetate extraction-flame photometry method. TN was extracted using the Kjeldahl nitrogen determination method, TP was extracted using the acid dissolution molybdenum antimony resistance colorimetric method, TK was extracted using acid dissolution flame photometry method. Trace elements in soil and plant specimens were analyzed using inductively coupled plasma mass spectrometry (ICP-MS, 7800, Agilent Technologies), Cl^-^ using ion chromatography (IC) method (Mishra et al., 2018).

#### 2.3.2 Agronomic traits

The agronomic traits of tobacco were measured according to the Chinese tobacco industry standard YC/T 142-1988.

The leaf area is calculated using the following formula:


$$\text{leaf area}({\text{cm}}^{2})=\text{leaf length}\times \text{leaf width}\times 0.6345.$$

#### 2.3.3 Biomass and nutrient content

Tobacco samples was dried at 105°C for half an hour and then dried at 70°C to constant weight to determine the biomass. Roots, stems, and upper leaves, middle leaves, lower leaves were made into analytical samples by grinding with a plant crusher, and then processing through a 0.5 mm plastic sieve.

The N content was determined using a Kjeldahl N analyze (Thilakarathna et al., 2019), the P content was analyzed via the molybdenum-antimony colorimetric method (Zhang et al., 2020), and the K content was measured with an FP640 flame photometer (Artyszak and Gozdowski, 2021).

The nutrient accumulation and fertilizer utilization rate are calculated using the following formula:


Nutrient accumulation(g/plant)=∑(nutrient content of each part×biomass of each part);

$$\begin{array}{c} \text{Fertilizer utilization rate}(\%)=(\text{amount of nutrients absorbed by crops in fertilization area}-\\ \text{amount of nutrients absorbed by crops in nutrient deficient area})/\text{amount of nutrients used}\times 100\% \end{array}$$

#### 2.3.4 SPAD value

The SPAD value of tobacco plants were measured by using the Spad-520 PLUS portable chlorophyll meter.

#### 2.3.5 Economic character

The economic characters of tobacco were calculated according to the national standard (GB2635-92).

#### 2.3.6 Physical properties and chemical composition

The physical properties and chemical composition were measured according to the Chinese tobacco industry standard YC/T 174-2010.

The leaf moisture proportion, opening degree, leaf midrib proportion and density of leaves are calculated using the following formula:


The leaf moisture proportion(%)=(fresh sample mass−dry sample mass)/fresh sample mass×100%

The opening degree(%)=(leaf width/leaf length)×100%,

The leaf midrib proportion(%)=(total mass of 10 main vein stems/total mass of 10 tobacco leaves)×100

$$\text{The density of leaf}(\text{g}/{\text{m}}^{2})=\text{sample weight}/(\text{number of leaf pieces}\times \text{single piece area})$$

Chemical compositions including total sugar, reducing sugar, total N, nicotine, water-soluble chlorine, starch, and protein were analyzed using an AA3 continuous flow analyzer, while K content was determined by flame spectrophotometry (Ruan et al., 2021).

### 2.4 Statistical analysis

Microsoft Excel 2019 software was used to sort out data, and they were statistically analyzed using analysis of variance (ANOVA) in SPSS version 22.0 software (IBM, Chicago, USA). The significant difference among treatments was calculated by the least significant difference (LSD) and was considered significant when p< 0.05. 

## 3 Results

### 3.1 The effect of silicon on tobacco growth and SPAD value

To investigate the impact of different Si fertilizer application rates on the growth of tobacco at different stages, agronomic traits including plant height, stem circumference, effective leaf number, maximum leaf length, and maximum leaf width were measured during the flower bud stage and button stage of tobacco. Results showed that, compared with S0, the plant height of S50, S100, and S200 during the flower bud stage of tobacco significantly increased by 6.05%, 16.57%, and 9.74%, respectively (**Table 2**). Similarly, during the button stage, this phenomenon also occurred. Compared with S0, the tobacco plant height significantly increased under Si fertilizer treatment, with increases of 15.38%, 16.35%, and 19%, respectively (**Table 2**). This indicated that Si fertilizer significantly increased the height of tobacco plants. It is worth noting that for tobacco leaves, under Si fertilizer treatment, the maximum leaf length and maximum leaf area of tobacco in the flower bud stage were significantly increased; interestingly, the maximum leaf length of tobacco during the button stage significantly increased by 7.78%, 9.6%, and 6.03%, respectively, while the maximum leaf area was showed the same trend (**Table 2**). Compared with S0, the maximum leaf width of S200 has significantly increased, with an increase of 7.12% and 15.57% in the flower bud stage and button stage, respectively (**Table 2**). This indicated that the application of Si fertilizer significantly promoted the growth of tobacco leaves. The effective number of tobacco leaves were increased, with significant increases of 10.20% and 13.33% in S50 and S100 during the bud stage, respectively (**Table 2**). However, Si fertilizer had no significant effect on the stem circumference of tobacco. In summary, Si fertilizer promotes the growth and development of tobacco leaves and plants.

**Table 2:** The effects of different application rates of silicon fertilizer on the agronomic traits of tobacco.

Treatments	Time	Plant height (cm)	Stem circumference (cm)	Effective number of leaves (leaves/plant)	Maximum leaf length (cm)	Maximum leaf width (cm)	Maximum leaf area (cm^2^)
S0	flower budding stage	84.32 ± 0.45d	6.44 ± 0.34a	15.00 ± 0.67b	50.73 ± 0.07c	24.44 ± 0.44b	786.87 ± 14.29b
S50		89.42 ± 1.95c	6.77 ± 0.27a	16.53 ± 0.24a	56.09 ± 0.96b	24.99 ± 0.47b	889.30 ± 31.07a
S100		98.29 ± 1.14a	6.84 ± 0.20a	17.00 ± 0.33a	58.74 ± 0.51a	24.50 ± 0.58b	913.04 ± 15.91a
S200		92.53 ± 1.16b	6.63 ± 0.29a	15.22 ± 0.19b	55.25 ± 1.22b	26.18 ± 0.66a	917.79 ± 33.46a
S0	button stage	89.96 ± 2.91b	6.79 ± 0.21a	16.67 ± 1.00a	53.47 ± 1.46b	22.22 ± 1.01b	748.60 ± 43.81b
S50		103.80 ± 3.90a	7.08 ± 0.39a	17.67 ± 1.00a	57.92 ± 1.20a	20.30 ± 0.52b	756.24 ± 25.91b
S100		104.67 ± 2.69a	6.87 ± 0.09a	17.00 ± 0.33a	58.90 ± 1.31a	21.04 ± 1.13b	786.62 ± 50.09b
S200		107.05 ± 8.59a	7.03 ± 0.25a	17.61 ± 0.35a	56.98 ± 2.08a	25.68 ± 1.92a	888.32 ± 64.52a

The data are the mean ± standard deviation of three replicates, and different letters after values in the same column indicate significant difference by ANOVA test (P < 0.05).

**Figure 3:**
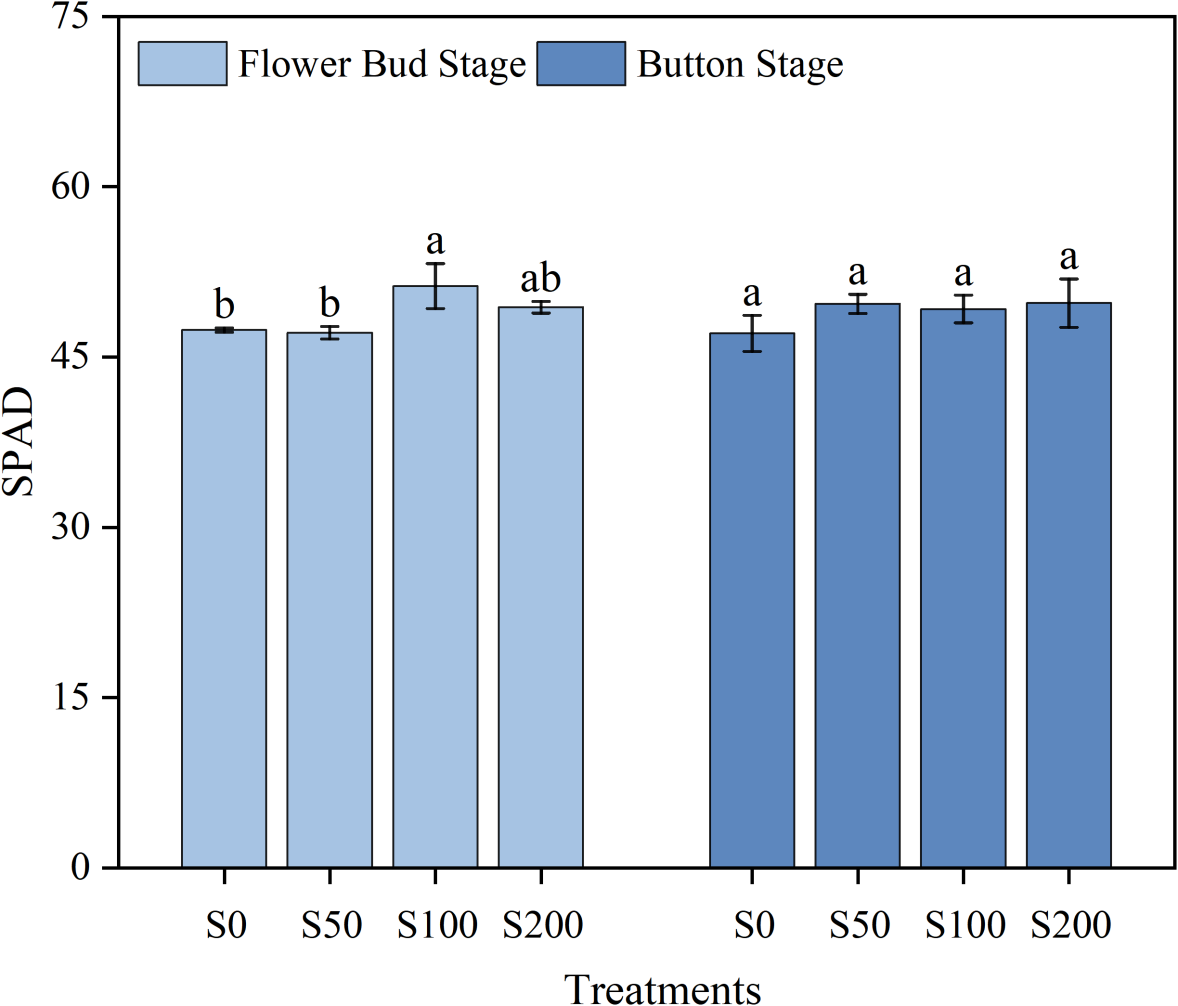
SPAD values in the different treatments. S0, conventional fertilization; S50, apply 750 kg of silicon fertilizer per hectare; S100, apply 1500 kg of silicon fertilizer per hectare; S200, apply 3000 kg of silicon fertilizer per hectare. The analysis of standard error and significant difference in the figure is based on the same variety. Data are means ± SE (n=4). During specific stage of tobacco, the significant difference among treatments is shown by different small letters on the error bars according to the least significant difference test (p< 0.05).

The SPAD value of tobacco leaves was measured during the bud stage and dome stage. The results indicated that, at the flower bud stage, compared with S0, Si improved the SPAD value of tobacco, and the SPAD value of tobacco leaves in S100 was significantly increased. At the button stage of tobacco, there was no significant difference among the treatments (**Figure 3**). Collectively, Si was significantly impacted the SPAD value of tobacco in the early stage.

### 3.2 The effect of silicon on tobacco biomass

During maturating stage of tobacco, the biomass of different parts of tobacco were measured, it indicated that Si fertilizer was significantly increasing the biomass of various parts of tobacco. Compared with the control, Si fertilizer significantly increased the biomass of tobacco plants, with S50, S100, and S200 significantly increasing by 26.53%, 19.95% and 19.50%, respectively (**Table 3**). Among the upper leaves, lower leaves, stems, and roots of S50 showed the highest growth, increasing by 17.74%, 20.35%, 28.31% and 48.91%, respectively (**Table 3**). It is interesting that the middle leaves showed the most significant increasing in S200, reaching 55.32g (**Table 3**). Overall, these results indicated that Si fertilizer significantly increased the biomass of various parts of tobacco.

**Table 3:** The effects of different application rates of silicon fertilizer on tobacco biomass.

Treatments	Time	Biomass of tobacco plants (g)						Proportion of plant biomass growth (%)					
		The whole plant	Upper leaves	Middle leaves	Lower leaves	Stem	Root	The whole plant	Upper leaves	Middle leaves	Lower leaves	Stem	Root
S0	maturating stage of tobacco	218.80 ± 4.84b	41.26 ± 1.22c	43.11 ± 0.43c	38.68 ± 2.74b	39.38 ± 5.08b	56.37 ± 6.92b	0	0	0	0	0	0
S50		276.84 ± 4.83a	48.58 ± 1.51a	47.24 ± 1.08b	46.55 ± 1.41a	50.53 ± 6.55a	83.93 ± 3.91a	26.53	17.74	9.57	20.35	28.31	48.91
S100		262.45 ± 5.47a	45.90 ± 0.68b	47.83 ± 1.52b	45.71 ± 1.82a	45.27 ± 5.05ab	77.75 ± 4.95a	19.95	11.25	10.94	18.17	14.94	37.94
S200		261.48 ± 17.85a	45.88 ± 0.67b	55.32 ± 2.77a	42.78 ± 2.00a	48.62 ± 2.40ab	68.88 ± 15.63ab	19.5	11.21	28.3	10.59	23.44	22.21

The data are the mean ± standard deviation of three replicates, and different letters after values in the same column indicate significant difference by ANOVA test (P < 0.05).

### 3.3 The effect of silicon on tobacco nutrient accumulation and fertilizer utilization efficiency

**Figure 4:**
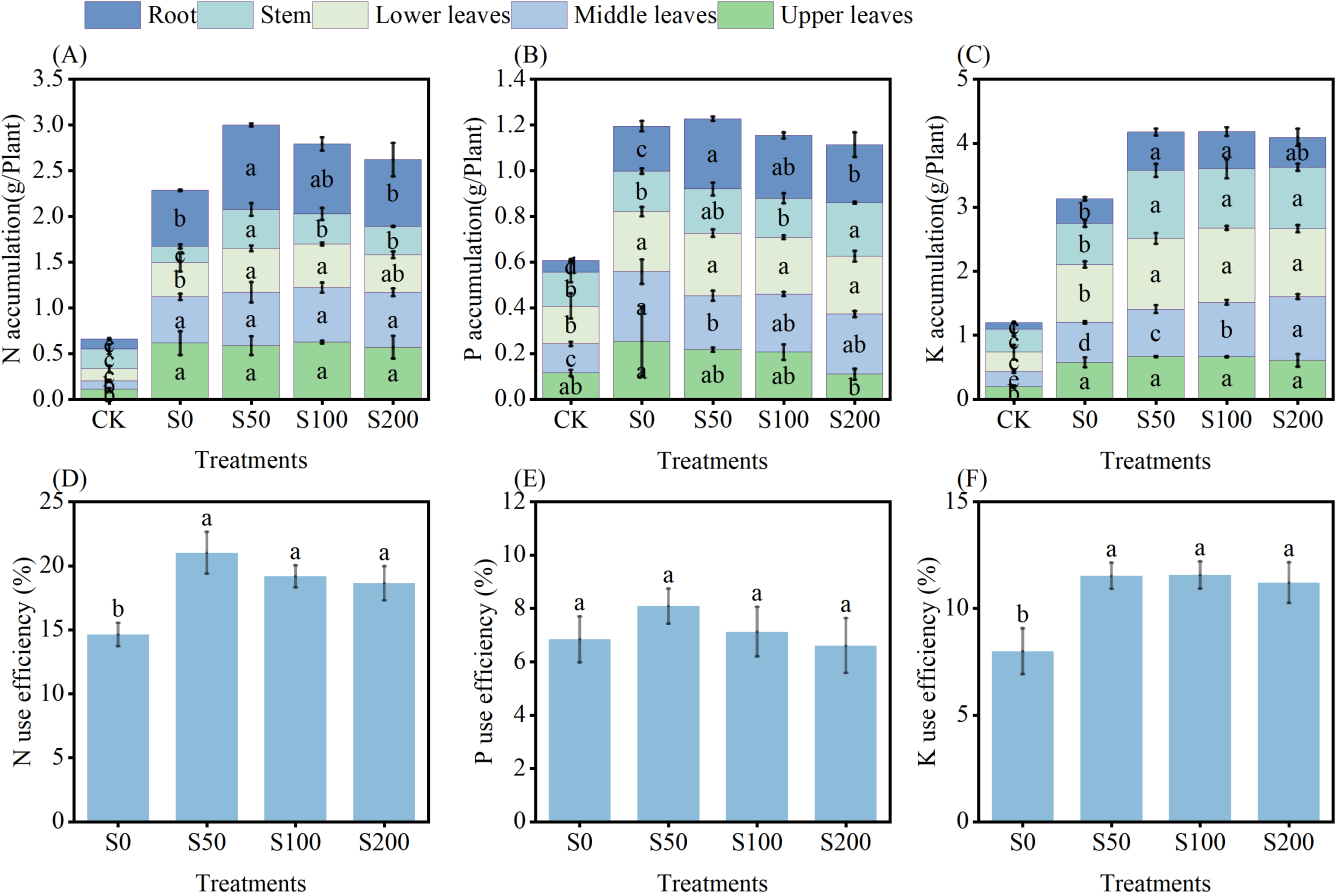
Nutrient accumulation and use efficiency in different treatments. **(A)** N accumulation; **(B)** P accumulation; **(C)** K accumulation; **(D)** N use efficiency; **(E)** P use efficiency; **(F)** K use efficiency. CK, blank, no fertilizer; S0, conventional fertilization; S50, apply 750 kg of silicon fertilizer per hectare; S100, apply 1500 kg of silicon fertilizer per hectare; S200, apply 3000 kg of silicon fertilizer per hectare. The analysis of standard error and significant difference in the figure is based on the same variety. Data are means ± SE (n=4). After specific days of transplantation, the different lowercase letters inside the stacked graph and above the error bar represent significant differences among treatments according to the least significant difference test (LSD; p< 0.05).

In order to investigate the effect of different Si fertilizer application rates on the nutrient content of tobacco, the accumulation of N, P, and K in various parts of tobacco was measured. These results indicated that compared with S0, the N accumulation in S50, S100 and S200 were increased by 31.14%, 22.16%, and 14.66%, respectively (**Figure 4A**). However, among Si treatment, the N accumulation of the lower leaves, stems, and roots in S50 showed the most significantly improvement, which were increased by 27.30%, 142.48%, and 50.15%, respectively (**Figure 4A**). This data indicated that Si was significantly influence the nitrogen content of tobacco, and the application rate of 750kg/ha Si fertilizer has the most significant effect on nitrogen accumulation in tobacco.

Research on tobacco P content found that compared with S0, there was no significant differences in P accumulation of plant and lower leaves in S50, S100, and S200 (**Figure 4B**). The P accumulation of roots in S50, S100 and S200 were increased by 56.04%, 38.98%, and 29.14%, respectively (**Figure 4B**). In contrast, the P accumulation of upper leaves of S200 were significantly decreased by 56.72%, while the P accumulation of stem of S200 were significantly increased by 32.13%; and on middle leaves, S50 was significantly decreased by22.66% (**Figure 4B**). These results indicated that Si fertilizer significantly changed P accumulation content of tobacco.

The application of Si significantly increased the K accumulation in various parts of the tobacco. Compared with S0, the K accumulation in S50, S100 and S200 were significantly increased by 28.08%, 28.30%, and 25.51%, respectively (**Figure 4C**). Interestingly, the K accumulation of upper leaves in S50, S100 and S200 were no significant difference, the K accumulation of middle leaves in S50, S100 and S200 were significantly increased by 18.86%, 36.18%, and 59.54%, respectively; similarly, the K accumulation of lower leaves and stem were significantly increased (**Figure 4C**). The K accumulation of roots in S50, S100 were significantly increased by 55.40%, 50.32%, and 20.83%, respectively, reaching0.59 g/plant and 0.58 g/plant (**Figure 4C**). In summary, the nutrient content of plants was significantly affected by Si fertilizer, and the application rate of moderate Si fertilizer (750–1500 kg/ha) is better.

To explore the impact on the fertilize use efficiency, the utilization efficiency of N, P and K were measured, we found that Si increased the fertilizer use efficiency. Compared to S0, the N use efficiency of S50, S100 and S200 were increased by 43.71%, 31.10%, and 27.42%, respectively (**Figure 4D**). And the K use efficiency of S50, S100 and S200 were improved by 44.25%, 44.63%, and 40.25%, respectively (**Figure 4F**). While there was no significant difference among the treatments in P use efficiency (**Figure 4E**).

### 3.4 The effect of silicon on tobacco economic characteristics

In order to elucidate the impact of Si on economic characteristics, five indicators including yield, output value, average price, proportion of high-quality tobacco leaves and proportion of medium to high-quality tobacco leaves were studied. The results showed that compared with S0, the yields of S50, S100 and S200 were increased by 15.70%, 13.32%, and 17.00% respectively; similarly, the output values were increased by 30.76%, 23.14%, and 21.39% respectively (**Table 4**). In addition, the proportion of high-quality tobacco leaves and medium to high-quality tobacco leaves were both the highest in S50, which were 80.11% and 94.75%, respectively, the average price of S50 was significantly increased by 13.08% compared to S0 (**Table 4**). These results indicated that Si increased the yield and output value of tobacco, improved economic benefits. Furthermore, the effect of moderate Si fertilizer application is better.

**Table 4:** The effects of different application rates of silicon fertilizer on the economic characteristics of tobacco.

Treatments	Yield(kg/hm^2^)	Output value(yuan/hm^2^)	Average price(yuan/kg)	Proportion of premium tobacco leaves (%)	Proportion of medium and high-quality tobacco leaves (%)
S0	2048.80 ± 73.10b	64227.74 ± 3216.53b	32.02 ± 0.07b	65.58	88.96
S50	2370.49 ± 66.76a	83986.71 ± 1964.59a	35.45 ± 1.40a	80.11	94.75
S100	2321.61 ± 5.53a	79089.54 ± 5009.04a	34.07 ± 2.21ab	80.29	93.48
S200	2397.19 ± 35.60a	77963.24 ± 956.35a	32.52 ± 0.09b	78.01	92.29

The data are the mean ± standard deviation of three replicates, and different letters after values in the same column indicate significant difference by ANOVA test (P < 0.05).

### 3.5 The effect of silicon on the physical properties of tobacco leaves

In order to study the effect of Si on tobacco quality, the physical properties of the upper, middle, and lower leaves of tobacco were tested, including single leaf weight, density of leaf, leaf moisture proportion, leaf midrib proportion, opening degree, thickness, leaf length, leaf width and leaf area. On upper leaves, compared with S0, the single leaf weight in S50, S100 and S200 were increased by 25.17%, 11.55%, and 10.86%, respectively; and the leaf length of tobacco were significantly increased by 7.99%, 8.84%, and 6.56%, respectively; but the density of leaves was decreased by 6.08%, 9.12%, and 18.80%, respectively, S200 was significantly decreased to 108.42 g/m^2^ (**Table 5**). Data showed that Si promoted the growth of upper tobacco leaves, reduce tobacco density, and improved tobacco quality. Compared with S0, the leaf midrib proportion in S50 and S100 were decreased by 10.53% and 2.40%, respectively, while the leaf midrib proportion of S200 was increased by 3.95% (**Table 5**). This indicate that high application of Si fertilizer actually increased the stem content of upper leaves, which is not conducive to improving the usability of tobacco leaves.

**Table 5:** The effects of different application rates of silicon fertilizer on the physical properties of tobacco.

Treatments	Different parts of tobacco leaves	Single leaf weight (g)	Density of leaf (g/m^2^)	Leaf moisture proportion (%)	Leaf midrib proportion (%)	Opening degree (%)	Thickness (mm)	Leaf length (cm)	Leaf width (cm)	Leaf area (cm^2^)
S0	Upper leaves	5.77 ± 0.40b	133.52 ± 11.77a	17.90 ± 11.53a	28.10 ± 1.72ab	26.23 ± 1.69a	0.20 ± 0.02a	46.73 ± 0.76b	12.27 ± 0.99a	364.04 ± 35.29a
S50		7.23 ± 0.58a	125.41 ± 16.18ab	11.56 ± 0.66a	25.14 ± 0.93b	28.07 ± 2.85a	0.19 ± 0.02a	50.47 ± 1.92a	14.20 ± 1.91a	456.17 ± 76.57a
S100		6.44 ± 0.64ab	121.35 ± 10.48ab	12.67 ± 1.53a	27.43 ± 1.49ab	27.37 ± 1.64a	0.18 ± 0.02a	50.87 ± 1.10a	13.93 ± 1.10a	450.17 ± 44.48a
S200		6.40 ± 0.26ab	108.42 ± 6.87b	14.54 ± 3.36a	29.21 ± 1.88a	26.63 ± 1.35a	0.17 ± 0.01a	49.80 ± 0.60a	13.27 ± 0.81a	419.38 ± 30.17a
S0	Middle leaves	6.41 ± 0.34a	104.27 ± 4.54a	13.72 ± 2.17a	26.17 ± 1.08a	32.61 ± 1.04a	0.15 ± 0.00a	51.33 ± 1.50a	16.73 ± 0.58b	545.20 ± 30.14a
S50		7.59 ± 0.92a	114.46 ± 5.98a	14.01 ± 2.71a	20.95 ± 1.39c	34.68 ± 2.79a	0.16 ± 0.01a	52.07 ± 2.70a	18.07 ± 1.94ab	598.38 ± 90.21a
S100		6.77 ± 0.98a	106.25 ± 10.34a	9.14 ± 5.14a	23.93 ± 1.28ab	33.62 ± 2.60a	0.15 ± 0.01a	52.60 ± 1.78a	17.67 ± 1.10ab	589.45 ± 38.68a
S200		7.28 ± 0.21a	105.02 ± 11.85a	13.00 ± 0.89a	23.26 ± 1.04b	35.95 ± 0.69a	0.15 ± 0.01a	53.40 ± 0.87a	19.20 ± 0.53a	650.68 ± 26.74a
S0	Lower leaves	5.88 ± 1.11a	73.51 ± 9.94a	14.47 ± 1.70a	27.00 ± 0.24b	38.84 ± 2.33a	0.11 ± 0.01a	50.20 ± 2.60b	19.47 ± 0.81a	620.21 ± 44.26a
S50		6.53 ± 0.66a	73.79 ± 4.10a	15.90 ± 1.39a	29.02 ± 1.36a	37.21 ± 2.02a	0.10 ± 0.00a	54.87 ± 2.31a	20.40 ± 1.06a	710.52 ± 54.84a
S100		5.71 ± 0.40a	65.68 ± 5.30a	14.28 ± 1.19a	30.38 ± 0.17a	37.79 ± 1.99a	0.10 ± 0.01a	52.27 ± 1.62ab	19.73 ± 0.64a	654.26 ± 23.92a
S200		6.49 ± 0.67a	74.17 ± 4.11a	15.14 ± 0.29a	29.81 ± 1.54a	38.58 ± 2.27a	0.10 ± 0.00a	53.93 ± 1.86ab	20.80 ± 1.20a	712.04 ± 52.39a

The data are the mean ± standard deviation of three replicates, and different letters after values in the same column indicate significant difference by ANOVA test (P < 0.05).

On middle leaves, compared with S0, the leaf midrib proportion of S50, S100 and S200 were decreased by 19.96%, 8.58%, and 11.12%, respectively; the leaf width was increased by 7.97%, 5.58%, and 14.74%, respectively, S200 was significantly increased to 19.20 cm; similarly, the leaf area was increased by 9.75%, 8.12%, and 19.35%, respectively (**Table 5**). This indicates that Si can promote the growth of tobacco leaves in the middle, reduce the stem content of tobacco leaves, and then increase yield.

The lower leaves of tobacco were impacted by Si. On lower leaves, compared with S0, the leaf midrib proportion of S50, S100 and S200 were increased by 7.48%, 12.50%, and 10.40%, respectively; the leaf length in S50, S100 and S200 were increased by 9.30%, 4.12%, and 7.44%, respectively, S50 was significantly increased to 54.87 cm; the leaf area in S50, S100 and S200 were increased by 14.56%, 5.49%, and 14.81%, respectively (**Table 5**). In summary, Si affected the physical properties of tobacco leaves, promoted the growth of tobacco leaves, and increased the availability of upper and middle leaves. However, high usage of Si fertilizer actually increased the leaf midrib proportion.

### 3.6 The effect of silicon on the chemical composition of tobacco leaves

Similarly, in order to investigate the effect of Si on the quality of tobacco, chemical indicators of tobacco leaf from different parts were tested. The results indicate that Si fertilizer improved the applicability of tobacco leaves. On the upper leaves, compared with S0, the content of reducing sugar in S50 was significantly increased by 15.21%, while S100 and S200 were significantly decreased by 13.92% and 21.93%, respectively; the K content of S50, S100 and S200 were significantly increased by 6.42%, 12.10%, and 11.56%, respectively; the chlorine content and the ratio of reducing sugar to total sugar of S50 were significantly increased by 20.44% and 19.09%, respectively; the nicotine content of S200 was significantly increased by 13.99%; while the total sugar, starch content and the ratio of total sugar to nicotine of S200 were significantly decreased by 14.29%, 12.05%, and 24.42%, respectively (**Table 6**). On the middle leaves, compared with S0, the K content of S100 and S200 were significantly increased by 21.60% and 24.40%, respectively; the nicotine of S50 was significantly increased by 18.56%, the chlorine content of S100 was significantly decreased by 11.79%; the total sugar, chlorine content and the ratio of K content to chlorine content of S200 were significantly increased by 15.50%, 26.72%, and 38.35%, respectively (**Table 6**). On the lower leaves, compared with S0, the K content of S100 was significant increased by 8.86%; the chlorine content of S50, S100 and S200 were significantly reduced by 22.25%, 18.57%, and 20.44%, respectively; the ratio of K content to chlorine content of S50, S100 and S200 were increased by 30.56%, 33.29% and 33.15% respectively; the reducing sugar content and the ratio of reducing sugar to total sugar of S50 were significantly increased by 23.96% and 15.05%, respectively; while the starch content of S50 was significantly decreased by 18.98%; the ratio of total sugar to nicotine of S100 was decreased by 7.52%; the N content of S200 was significantly decreased by 15.26%, while the starch content of S200 was significantly increased by 15.14% (**Table 6**).

**Table 6:** The effect of different application rates of silicon fertilizer on the chemical composition of tobacco.

Treatments	Different parts of tobacco leaves	Nitrogen (%)	Nicotine (%)	Total sugar (%)	Reducing sugar (%)	Starch (%)	Potassium (%)	Chlorine (%)	Protein (%)	Total sugar/Nicotine	Potassium/Chloride	Nitrogen/Nicotine	Reducing sugar/Total sugar
S0	Upper leaves	1.37 ± 0.10a	2.81 ± 0.05b	27.85 ± 0.28a	18.80 ± 0.55b	2.81 ± 0.22a	1.29 ± 0.06b	0.29 ± 0.02b	6.51 ± 1.68a	9.93 ± 0.07a	4.44 ± 0.51ab	0.49 ± 0.04a	0.68 ± 0.03b
S50		1.20 ± 0.17a	2.65 ± 0.05b	26.90 ± 1.21a	21.64 ± 1.97a	2.63 ± 0.16ab	1.37 ± 0.04a	0.35 ± 0.01a	5.04 ± 1.08a	10.17 ± 0.64a	3.91 ± 0.27b	0.46 ± 0.07a	0.80 ± 0.05a
S100		1.36 ± 0.04a	2.75 ± 0.15b	25.85 ± 1.84ab	16.18 ± 0.41c	2.88 ± 0.04a	1.45 ± 0.02a	0.31 ± 0.01ab	5.45 ± 0.69a	9.40 ± 0.42a	4.60 ± 0.17a	0.50 ± 0.01a	0.63 ± 0.03b
S200		1.39 ± 0.00a	3.20 ± 0.00a	24.00 ± 1.70b	14.68 ± 1.22c	2.47 ± 0.13b	1.44 ± 0.03a	0.32 ± 0.03b	4.63 ± 1.54a	7.50 ± 0.52b	4.53 ± 0.29ab	0.43 ± 0.01a	0.61 ± 0.05b
S0	Middle leaves	1.17 ± 0.07a	1.73 ± 0.07b	27.99 ± 1.79a	24.68 ± 1.43a	2.62 ± 0.19b	1.45 ± 0.04b	0.27 ± 0.01b	5.48 ± 0.49a	16.21 ± 0.41ab	5.31 ± 0.10bc	0.68 ± 0.06a	0.88 ± 0.07a
S50		1.24 ± 0.25a	2.05 ± 0.09a	28.05 ± 1.71a	20.99 ± 6.08a	2.45 ± 0.01b	1.62 ± 0.04b	0.28 ± 0.01b	6.10 ± 1.30a	13.75 ± 1.41b	5.85 ± 0.15b	0.60 ± 0.11a	0.74 ± 0.17a
S100		1.20 ± 0.06a	1.68 ± 0.04b	29.37 ± 4.43a	24.68 ± 3.91a	2.62 ± 0.10b	1.77 ± 0.03a	0.24 ± 0.02c	6.44 ± 1.12a	17.44 ± 2.59a	7.35 ± 0.56a	0.71 ± 0.03a	0.84 ± 0.01a
S200		1.09 ± 0.06a	1.62 ± 0.08b	31.09 ± 0.64a	27.28 ± 0.91a	3.02 ± 0.08a	1.81 ± 0.03a	0.35 ± 0.00a	5.43 ± 0.40a	19.26 ± 0.93a	5.22 ± 0.12c	0.67 ± 0.02a	0.88 ± 0.03a
S0	Lower leaves	1.10 ± 0.04a	1.65 ± 0.06a	30.81 ± 2.53a	21.25 ± 2.66b	2.93 ± 0.06b	2.33 ± 0.07b	0.37 ± 0.02a	4.72 ± 1.42a	18.68 ± 1.11ab	6.34 ± 0.50b	0.67 ± 0.00ab	0.69 ± 0.03b
S50		1.05 ± 0.02a	1.50 ± 0.02a	31.42 ± 2.02a	26.34 ± 3.47a	2.38 ± 0.22c	2.37 ± 0.11b	0.29 ± 0.03b	5.31 ± 0.30a	20.90 ± 1.14a	8.27 ± 0.55a	0.70 ± 0.01a	0.84 ± 0.06a
S100		1.06 ± 0.06a	1.66 ± 0.16a	28.71 ± 3.89a	18.63 ± 1.72b	3.10 ± 0.09ab	2.54 ± 0.07a	0.30 ± 0.01b	5.17 ± 0.48a	17.27 ± 1.10b	8.45 ± 0.21a	0.64 ± 0.08ab	0.65 ± 0.03b
S200		0.93 ± 0.04b	1.58 ± 0.21a	32.13 ± 0.58a	23.49 ± 1.61ab	3.38 ± 0.30a	2.48 ± 0.05ab	0.29 ± 0.01b	4.68 ± 0.37a	20.61 ± 2.80ab	8.44 ± 0.02a	0.60 ± 0.05b	0.73 ± 0.04b

The data are the mean ± standard deviation of three replicates, and different letters after values in the same column indicate significant difference by ANOVA test (P < 0.05).

## 4 Discussion

### 4.1 Tobacco growth

Chlorophyll, as an essential pigment for photosynthesis, directly affects leaf photosynthetic capacity and organic matter accumulation (Zhang et al., 2018). Studies indicated that SPAD values were positively correlated with chlorophyll content and dry matter accumulation (Condorelli et al., 2018). Marxen et al. (2016) demonstrated that Si enhanced light interaction in plants. Researchers showed that Si application in rice and maize increased the maximum leaf area, aboveground dry matter weight and yields (Pati et al., 2016; Biswal et al., 2024.). In this study, the plant height and maximum leaf area of tobacco were increased under silicon treatment (**Table 2**).

This study confirmed that Si fertilizer treatment significantly increased the SPAD values and biomass of tobacco (**Figure 3**; **Table 3**). At the flower bud stage, the S100 exhibited significantly higher SPAD values than other treatments, although no significant differences were observed among the remaining treatments, their SPAD values remained numerically higher than S0 (**Figure 3**). That indicated that moderate Si supplementation improved photosynthetic capacity by elevating chlorophyll content, improved tobacco growth, the plants height and leaf area were significantly increased (Huang et al., 2024). This effect may be attributed to silicon’s ability to optimize light utilization through increased leaf conductivity (Verma et al., 2020b). Besides, moderate Si application promoted systemic growth by stimulating root development and nutrient absorption (Parecido et al., 2025). The whole plant biomass in S50, S100 and S200 were significantly increased by 26.53%, 19.95%, and 19.50%, respectively (**Table 3**). However, at the button stage of tobacco, there was no significant differences in SPAD values, which likely due to natural leaf senescence during later growth stages, which reduced silicon’s chlorophyll synthesis benefits (Luyckx et al., 2017). In addition, excessive application of Si fertilizer may lead to thicker leaves, decreased stomatal conductance, affected CO_2_ absorption and photosynthetic efficiency, decreased SPAD values, poor root development and stunted plants (Huang et al., 2024). But this phenomenon was not found in this study (**Figure 3**).

### 4.2 Nutrient use efficiency

#### 4.2.1 Nitrogen

It demonstrated that the application of Si enhanced N availability, promoted plant N uptake, and N use efficiency (da Silva et al., 2023; Neu et al., 2017). The results of this study were similar (**Figure 4A**). The application of Si promoted the N metabolism related microorganisms in the soil, thereby improving N availability, which was beneficial for plants to absorb N from the soil (Liang et al., 2021). Furthermore, Si induces the expression of NO3^-^ transporter in roots, thereby improving N absorption and use efficiency, (Neu et al., 2017; Zou et al., 2018), finally, increased the accumulation of N in plants (**Figure 4A**). Similarly, in this study, the N use efficiency was significantly increased by application of Si (**Figure 4D**).

#### 4.2.2 Phosphorus

The application of Si fertilizer at a rate of 750 kg/ha increased the P accumulation in tobacco (**Figure 4B**), similar results was observed in wheat (Triticum aestivum cv. Benchmark) and maize (Gunnarsen et al., 2022). The application of Si fertilizer reduced the soil’s P retention capacity, leading to an increase in the level of water-soluble P thereby increasing the available P content in the soil (Alsaeedi et al., 2019). Researchers found that Si application increased the activity of functional microorganisms and important metabolic enzymes related to P cycling in the soil, thereby promoting P mineralization and improving effectiveness (Liang et al., 2021).

The P content of winter wheat varies with changes in Si supply (Hang et al., 2024). Liang et al. (2021)found that with the increased of Si, the activities of acid phosphatase (AcPA) in soil first increase and then decrease, when the amount of Si added is below a certain threshold, it had a positive impact on plant growth and soil nutrient utilization, however, when the threshold was exceeded, high levels of Si may lead to stress responses that negatively effects on plant growth and soil nutrient utilization. That is similar to this study, with the increase of Si fertilizer application, the P accumulation and P use efficiency were first increased and then decreased (**Figures 4B, E**). Hu et al. (2018) found that when Si accumulates excessively in plant, it reduces the uptake of P. In this study, the P accumulation was reduced in S100 and S200 treatments, and the P content in the middle leaves of S200 treatment was significantly lower than that S0 (**Figure 4B**). This phenomenon may be attributed to two distinct mechanisms: (1) Si reduces P uptake by downregulating the expression of the P transporter gene (Hu et al., 2018), (2) excessive Si reduces P availability in the soil (Schaller et al., 2021).

#### 4.2.3 Potassium

Numerous studies have demonstrated that Si application significantly enhanced K use efficiency and K accumulation in plants (Barreto et al., 2022; Parecido et al., 2025). Consistent with these findings, our study revealed similar trends (**Figure 4**). Specifically, K use efficiency increased by 44.25% in the S50 compared to the S0 (**Figure 4F**). This improvement may be attributed to silicon’s role in stabilizing cellular membranes and optimizing K transport channels (Ghosh et al., 2022). On the one hand, Si enhances K uptake by increased of aquaporin gene expression and aquaporin activity in plant tissues; on the other hand, the genes responsible for K transport from root cortical cells to the xylem were activated, thereby improving long-distance K mobilization and facilitating K translocation (Barreto et al., 2022). These synergistic mechanisms collectively explain the observed increase in K content in tobacco leaves following Si fertilizer application.

### 4.3 Tobacco quality

#### 4.3.1 Physical properties

Si extends leaf growth period and promotes leaf growth by synthesizing cytokinins (Etesami et al., 2021). The study showed that the application of Si fertilizer within 750–1500 kg/ha increased the single leaf weight of tobacco, and then, increased the yields (**Tables 4**, **5**). However, when the application rates reached 3000 kg/ha, negative impact on single leaf weight was observed (**Table 5**). Moderate Si application promotes cell wall thickening and leaf cell expansion, thereby increasing single-leaf weight in tobacco (Verma et al., 2020b). In contrast, excessive Si induces over-lignification of the cell wall, which restricts cellular enlargement and ultimately reduces leaf biomass (Coskun et al., 2019). Si optimizes the structure of tobacco leaves by reducing the deposition of lignin in the main veins, while excessive Si leads to an increased degree of lignification in the main veins, the leaf midrib proportion of S50 leaves was the lowest, while the leaf midrib proportion of S200 was increased (Kumari et al., 2022; El-Saadony et al., 2022) (**Table 5**). In addition, excessive Si induced excessive enhancement of stem mechanical strength (Schaller et al., 2021), as evidenced by the increased leaf midrib proportion in the lower leaves of S200 (**Table 5**).

#### 4.3.2 Chemical composition

In this study, the effects of different amounts of Si fertilizer on different parts of tobacco leaves was vary (**Table 6**). Si fertilization enhanced sugar conversion, improving leaf sweetness and aroma, whereas excessive application inhibited sugar-degrading enzyme activity, reducing sugar accumulation and conversion efficiency (Luyckx et al., 2017). Our results align with these findings, the upper leaves of S50 exhibited the highest reducing sugar content, correlating with optimal flavor characteristics, while the lower leaves of S200 accumulated more total sugar but displayed reduced conversion efficiency (**Table 6**). These results responses highlight the different effects of Si application rates on leaf biochemistry.

Luyckx et al. (2017) established that appropriate Si optimizes the sugar-nicotine balance, reducing leaf harshness. Our data confirm this, with S50 achieved an ideal sugar-alkaloid ratio of 10.17 in upper leaves, whereas the 3000 kg/ha application significantly decreased this ratio while increasing nicotine content (**Table 6**), which potentially exacerbating smoking harshness.

Si suppressed the chloride channels (CLC family), resulted in reduced chlorine levels across Si treatments (S50, S100 and S200) relative to the control (S0) (Kumari et al., 2022). Notably, although S200 displayed marginally elevated K content, but the K-to-chlorine ratio of S100 was the more suitable value (**Table 6**), which was attributable to excessive Si may lead to chloride ion accumulation due to soil acidification (Schaller et al., 2021). This chemical content shift coincided with decreased protein content in silicon-treated leaves (**Table 6**), which indicating a metabolic change from N to C assimilation pathways (Huang et al., 2024). Collectively, Si fertilizer affected the quality of tobacco, and moderate Si fertilizer improved the quality of tobacco.

### 4.4 Economic characteristics

A large number of researchers found that the application of Si increased the yields of corn, cotton, rice and sugarcane (Yan et al., 2021; Li et al., 2018; Sakurai et al., 2015; Verma et al., 2020a). In this study, the application of Si increased the yields of tobacco as well, which mainly due to Si promoted the growth of tobacco and increased its biomass (**Table 4**). Additionally, moderate Si improved the physical properties and chemical balance of tobacco leaves, thus enhanced industrial usability, and the proportion of medium to high-quality tobacco was increased, besides, the proportion of medium to high-quality tobacco in the S50 treatment reached 94.75%, which increased the average price of tobacco leaves (**Table 4**). The increase of production and average price which together lead to an increase in output value, which is beneficial for the income of tobacco farmers and the development of the industry. However, excessive investment in Si may weaken this benefit.

## 5 Conclusion

Our study demonstrated that moderate Si application rates (750–1500 kg/ha) significantly improved tobacco productivity and quality. Si application at 750–1500 kg/ha increased the whole-plant biomass increased by 19.95%-26.53%, nutrient use efficiency was improved 34.83%-41.42%, high-quality leaves proportion was increased by 14.53%-14.71%, economic output value was boosted by 23.14%-30.76%. Among them, the S50 performed the best. However, excessive Si application (3000 kg/ha, S200) increased the leaf midrib proportion in the lower leaves by 10.40% and chemical imbalances was emerged, resulting in reduced of industrial availability. The study underscores the necessity of precision Si management to sustainably optimize tobacco quality, providing a scientific basis for Si fertilizer application in tobacco cultivation with practical guidance significance.

## Data Availability

The original contributions presented in the study are included in the article/supplementary material. Further inquiries can be directed to the corresponding author.
